# Cardiac affection associated to severe Multisystem Inflammatory Syndrome in Children (MIS-C) in a 6-year-old girl with a single coronary artery

**DOI:** 10.1007/s00392-022-02060-9

**Published:** 2022-07-29

**Authors:** Jochen Pfeifer, Peter Fries, Lorenz Thurner, Hashim Abdul-Khaliq

**Affiliations:** 1 Department of Pediatric Cardiology, Saarland University Medical Center, Kirrberger Strasse, 66421 Homburg, Germany; 2 Department of Diagnostic and Interventional Radiology, Saarland University Medical Center, Homburg, Germany; 3 José Carreras Center for Immuno and Gene Therapy and Department of Internal Medicine I, Saarland University Medical Center, Homburg, Germany


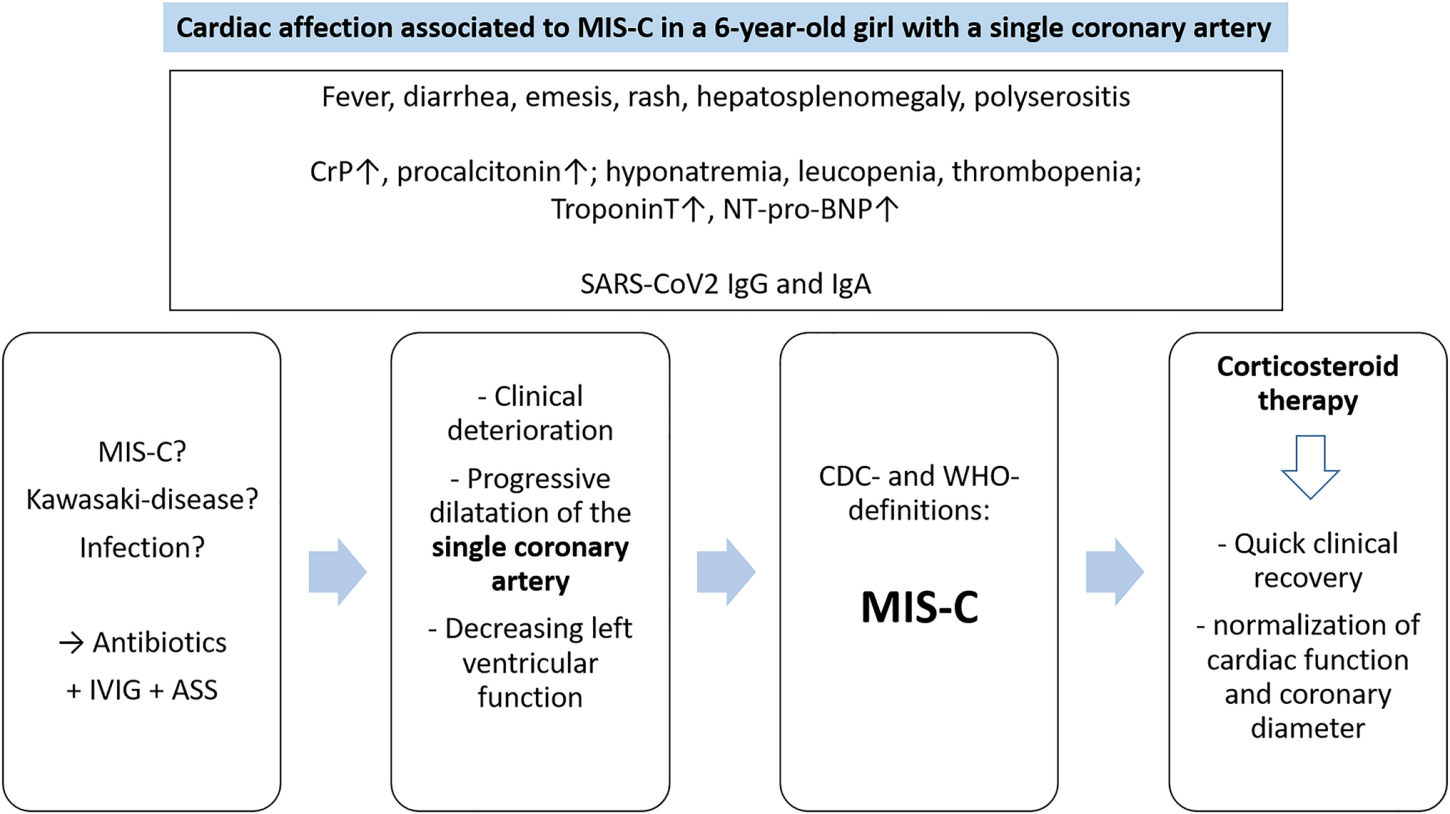
Sirs:

A 6-year-old former healthy girl was admitted to hospital 4 weeks after asymptomatic SARS-CoV2 infection. She presented in a poor general condition with fever for 5 days, diarrhea, emesis, rash, and hepatosplenomegaly. Laboratory examination showed hyponatremia, elevated inflammatory parameters, thrombopenia, and increasing cardiac parameters in terms of elevation of troponin T and pro-brain natriuretic peptide (NT-pro-BNP) as well as anemia and leucopenia (the Table 1 shows the laboratory findings in the course of the therapy). Serological examination revealed SARS-CoV-2-antibodies (both IgG and IgA).

**Table 1:** Laboratory characteristics of the patient

Laboratory analysis	Reference value	Day 1	Day 3	Day 6	Day 13	Follow up after 6 months
Hemoglobin	10.8–15.6 g/dl	8.5	8.4	7.7	13.8	10.8
Leucocytes	4800–12,000/µl	3400	24,900	13,200	14,700	5000
Platelets	186,000–488,000/µl	100,000	120,000	150,000	778,000	282,000
Sodium (Na^+^)	135–145 mmol/l	129	141	139	137	141
Potassium (K^+^)	3.4–5.1 mmol/l	3.4	3.8	4.6	5.3	4.4
Creatinine	0.4–0.6 mg/dl	0.6	0.9	0.7	0.4	0.6
AST	10–40 U/l	35	27	10	35	33
ALT	5–25 U/l	17	14	7	40	20
Troponin T	< 14 pg/ml	< 3	45	32	19	< 3
NT-pro-BNP	< 190 pg/ml	2069	28,667	52,003	493	163
Albumine	38–54 mg/l	26	45	51	na	na
Ferritine	7–84 ng/ml	421	459	na	na	na
CrP	< 5 mg/l	184	182	13.8	1.5	< 0.6
Procalcitonin	< 0.05 ng/ml	6.3	63.2	4.6	na	na
D-Dimer	< 0.5 mg/l	3.7	4.0	2.0	na	na

*AST* aspartate aminotransferase, *ALT* alanine aminotransferase, *NT-pro-BNP* N-terminal pro-brain natriuretic peptide, *CrP* C-reactive protein

Because both severe infection and Kawasaki disease (KD) initially were possible differential diagnoses, an antibiotic treatment was started, as well as high-dose ASS (30 mg/kg/d) and intravenous immunoglobulin (IVIG, 2 g/kg) were administered. After developing a shock symptomatic and polyserositis, the child was transferred to pediatric ICU. Pleural and peritoneal draining as well as inotropic and diuretic therapy were necessary. Non-invasive ventilation had to be performed.

**Fig. 1:**
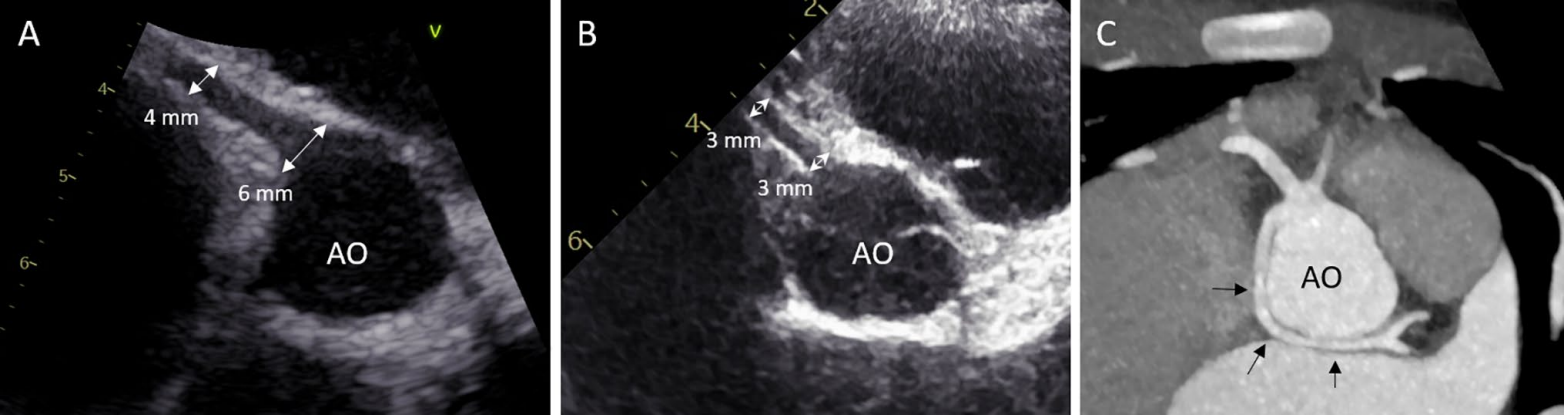
**A** transthoracic echocardiographic image at day 3: aortic root (AO) with a single coronary artery arising from the right aortic sinus; diameter 6 mm at ostium and 4 mm distally (white arrows). **B** transthoracic echocardiographic image at follow-up: diameters now decreased to 3 mm both at ostium and distally (white arrows). **C** computed tomography image: aortic root (AO) with a single coronary artery arising from the right aortic sinus and a left coronary trunk with retroaortic course (black arrows)

Echocardiography initially showed mitral regurgitation and decreased systolic function of the left ventricle (EF < 50%). Furthermore, there was a single coronary artery deriving from the right aortic sinus as an incidental finding. During daily echocardiographic controls, the ostial coronary diameter increased from 2.5 up to 6 mm within the first 3 days (Fig. 1). ECG showed non-specific abnormal repolarization in terms of flattened T waves.

After exclusion of viral or bacterial infection, Multisystem Inflammatory Syndrome in Children (MIS-C) was the hypothesized diagnosis according to the CDC and WHO definitions [1]. In a refractory state, the girl was then treated by administration of intravenous pulse methylprednisolone (30 mg/kg/d for 5 days, followed by prednisolone 2 mg/kg/d with gradual tapering) whereupon crucial improvement occurred with regressing clinical symptoms and defervescence within the next days. The echocardiographic findings including systolic function and coronary diameter (Fig. 1) as well as the blood parameters improved and nearly normalized. Interestingly, the cardiac markers gradually decreased and reached normal levels within 6 months (Table 1). Discharge from hospital was possible at day 16 with oral anticongestive medication and ASS.

Cardiac computed tomography confirmed the single coronary artery arising from the right coronary sinus. The branching left coronary artery showed a retroaortic course (Fig. 1). According to the Lipton classification, this represents a type RII-P single coronary artery [2]. There were no signs of acute myocarditis in cardiac magnetic resonance tomography.

MIS-C is a rare complication following SARS-CoV-2 infection, usually occurring after an interval of 2–6 weeks [1]. The clinical manifestations may be similar to those of KD including affection of coronary arteries in 6–24% [3, 4]; in KD coronary localized single or multiple aneuryms occur in about 25%. Macrophage activation syndrome, toxic shock syndrome, sepsis, and other inflammatory or infectious diseases are differential diagnoses to be considered [5]. To date, the pathogenesis of MIS-C is only partially understood; the role of autoantibodies and cytokine mediated inflammation is discussed in several studies [6, 7]. Recently, we reported on neutralizing autoantibodies against the interleukin 1-receptor antagonist (IL-1Ra-Ab) in MIS-C [8] which as well had been detected in the initial samples of this patient. They may possibly play a key role in the MIS-C associated hyperinflammation, including affection of small systemic and coronary vessels.

In cases of severe MIS-C, administration of methylprednisolone and IVIG is recommended [1]. In refractory states, anakinra (a recombinant interleukin 1-receptor antagonist) may be considered [1].

An isolated single coronary artery is a very rare congenital variant with an incidence of about 0.024–0.066% [9]. There are neither standard values nor z scores for diameters of single coronary arteries available. However, the rapid dynamic changes of the arterial diameter during the clinical course was suggestive for pathological ectasia of the main coronary vessel. Notably, the coronary artery was longitudinally enlarged; neither circumscribed saccular or regional aneurysms (as typical for KD) nor myocardial ischemia did occur. Another difference is the prompt return to normal coronary diameter as KD associated aneurysms >/= 6 mm use to diminish within several months or years [10, 11]. Patients with giant aneurysms (> 8 mm) are at highest risk for cardiac events. These different manifestations may be caused by different immunological inflammatory pathogeneses of both diseases [8].

Strict cardiac evaluation and clinical surveillance are necessary in patients suffering from MIS-C. In ultra-rare cases of combined congenital and acquired coronary affections, the risk for myocardial ischemia is incalculable.
